# Area-level and individual correlates of active transportation among adults in Germany: A population-based multilevel study

**DOI:** 10.1038/s41598-019-52888-x

**Published:** 2019-11-08

**Authors:** J. D. Finger, G. Varnaccia, L. Gabrys, J. Hoebel, L. E. Kroll, S. Krug, K. Manz, S. E. Baumeister, G. B. M. Mensink, C. Lange, M. F. Leitzmann

**Affiliations:** 1 Department of Epidemiology and Health Monitoring, Robert Koch Institute, Berlin, Germany; 2 Department of Sport and Prevention, University of Applied Sciences for Sport and Management Potsdam, Potsdam, Germany; 3 Chair of Epidemiology, Ludwig-Maximilian-University Munich at University Medicine Augsburg, Augsburg, Germany; 4 Independent Research Group Clinical Epidemiology, Helmholtz Zentrum München, German Research Center for Environmental Health, Neuherberg, Germany; 5 Department of Epidemiology and Preventive Medicine, University of Regensburg, Regensburg, Germany

**Keywords:** Risk factors, Ecological epidemiology

## Abstract

This study aimed at estimating the prevalence in adults of complying with the aerobic physical activity (PA) recommendation through transportation-related walking and cycling. Furthermore, potential determinants of transportation-related PA recommendation compliance were investigated. 10,872 men and 13,144 women aged 18 years or older participated in the cross-sectional ‘German Health Update 2014/15 – EHIS’ in Germany. Transportation-related walking and cycling were assessed using the European Health Interview Survey-Physical Activity Questionnaire. Three outcome indicators were constructed: walking, cycling, and total active transportation (≥600 metabolic equivalent, MET-min/week). Associations were analyzed using multilevel regression analysis. Forty-two percent of men and 39% of women achieved ≥600 MET-min/week with total active transportation. The corresponding percentages for walking were 27% and 28% and for cycling 17% and 13%, respectively. Higher population density, older age, lower income, higher work-related and leisure-time PA, not being obese, and better self-perceived health were positively associated with transportation-related walking and cycling and total active transportation among both men and women. The promotion of walking and cycling among inactive people has great potential to increase PA in the general adult population and to comply with PA recommendations. Several correlates of active transportation were identified which should be considered when planning public health policies and interventions.

## Introduction

Physical activity (PA) is one of the key modifiable health behaviors that needs to be promoted to reduce the epidemic burden of non-communicable diseases^1^. Physical activity takes place in different domains – at work, during active transportation, and in leisure time. Walking and cycling, the basic means of active transportation, comply with the definition of aerobic PA, the central aspect of the ‘Global Recommendations on Physical Activity for Health’ of the World Health Organization (WHO)^2^. Research on enhancing active transportation participation is needed because of health benefits of this particular PA domain^3,4^. Boosting active transportation is also ecologically meaningful as it reduces car traffic and contributes to combating climate change, reaching the targets of the Paris climate agreement and reducing air pollution in urban places^5^.

Increasing participation in active transportation has great potential to increase the share of population reaching the aerobic PA recommendations for health. In 2015, 77% of the German population lived and worked in urban places, and half of the distances travelled by car were shorter than 5 km^6^, indicating that this group could ideally commute to their workplaces by means of active transportation. The urbanized proportion is even higher in France (79%) and the United Kingdom (91%), and further significant growth in this area is expected for all countries by 2030^6^. A study among Dutch adults showed that 43% engaged in active transportation and reported an average of 24 and 28 minutes of daily PA through walking and cycling, respectively, which is more than the recommended minimum level of PA^7^. This finding suggests that there is considerable potential for reaching the aerobic PA recommendations with active transportation.

At present, it is unclear how many adults in Germany reach the PA recommendation through transportation-related PA. Moreover, additional evidence on the determinants of transportation-related PA recommendation compliance is needed to tailor effective interventions.

The current study aimed to estimate the prevalence of complying with the minimum level of recommended aerobic PA through transportation-related walking and cycling among adults in Germany. Furthermore, correlates of transportation-related PA recommendation compliance among adults were investigated.

## Methods

### Study design, setting, and participants

The German Health Update 2014–2015 (GEDA 2014/2015-EHIS) was a national health interview survey which implemented the European Health Interview Survey - wave 2 (EHIS2) in Germany^8^ and contained a domain-specific PA questionnaire that enables examining potential determinants of active transportation at the population level^8,9^. EHIS2 is a large-scale, cross-sectional health survey which was implemented by national governmental authorities in all 28 European Union (EU) Member States, Iceland and Norway between 2013 and 2015 according to uniform requirements^10^. All methods were performed in accordance with the relevant guidelines and regulations. Participation was compulsory for EU countries and was administrated on the basis of EU legislation^11^. The study design and methodology of implementing the EHIS2 in Germany is described in detail elsewhere^8^. Briefly, the GEDA 2014/2015-EHIS was conducted in Germany from 2014 to 2015. The study population comprised persons aged 15 years or older with permanent residency in Germany. The study sample was drawn based on a two-stage cluster sampling approach. In the first stage, 301 communities were randomly selected and then a gross sample of address data of 92,771 persons was provided by local population registers using age-stratified random sampling selection. A total of 24,016 persons (10,872 men and 13,144 women) participated in GEDA 2014/2015-EHIS; the response proportion was 27.6%^8^. The data were collected using self-administered postal paper or online web-based questionnaires.

### Definition of variables

*Transportation-related PA* outcome variables were assessed using the domain-specific EHIS-Physical Activity Questionnaire (EHIS-PAQ). The EHIS-PAQ questions, the development process, and validity and reliability information have been published in detail^9,12,13^. The EHIS-PAQ transportation-related PA index showed moderate-to-strong reliability, with a Spearman correlation coefficient of 0.72, and a Spearman correlation coefficient of 0.62 for validity when compared with PA during transportation as assessed by the International Physical Activity Questionnaire-Long Form (IPAQ-LF)^12^. Transportation-related walking and cycling were assessed with separate questions using similar wording: ‘In a typical week, on how many days do you walk/bicycle for at least 10 minutes continuously to get to and from places?’ and ‘How much time do you spend walking/bicycling in order to get to and from places on a typical day?’. The metabolic equivalent (MET) values used for computing MET-minutes were 3.3 for walking and 6.0 for cycling according to the IPAQ-LF manual^14^. One MET corresponds to the energy expenditure at a state of complete rest^15^. Achievement of the minimum recommended level of PA was defined as achieving at least 600 MET-min/week, which is in line with the definition of ‘sufficient PA’ according to the WHO^16^. Three binary variables were constructed: (a) *walking* ≥*600 MET-min/week*, (b) *cycling ≥600 MET-min/week* and (c) a *total active transportation* composite index ≥*600 MET-min/week* with the categories: yes/no.

**Figure 1:**
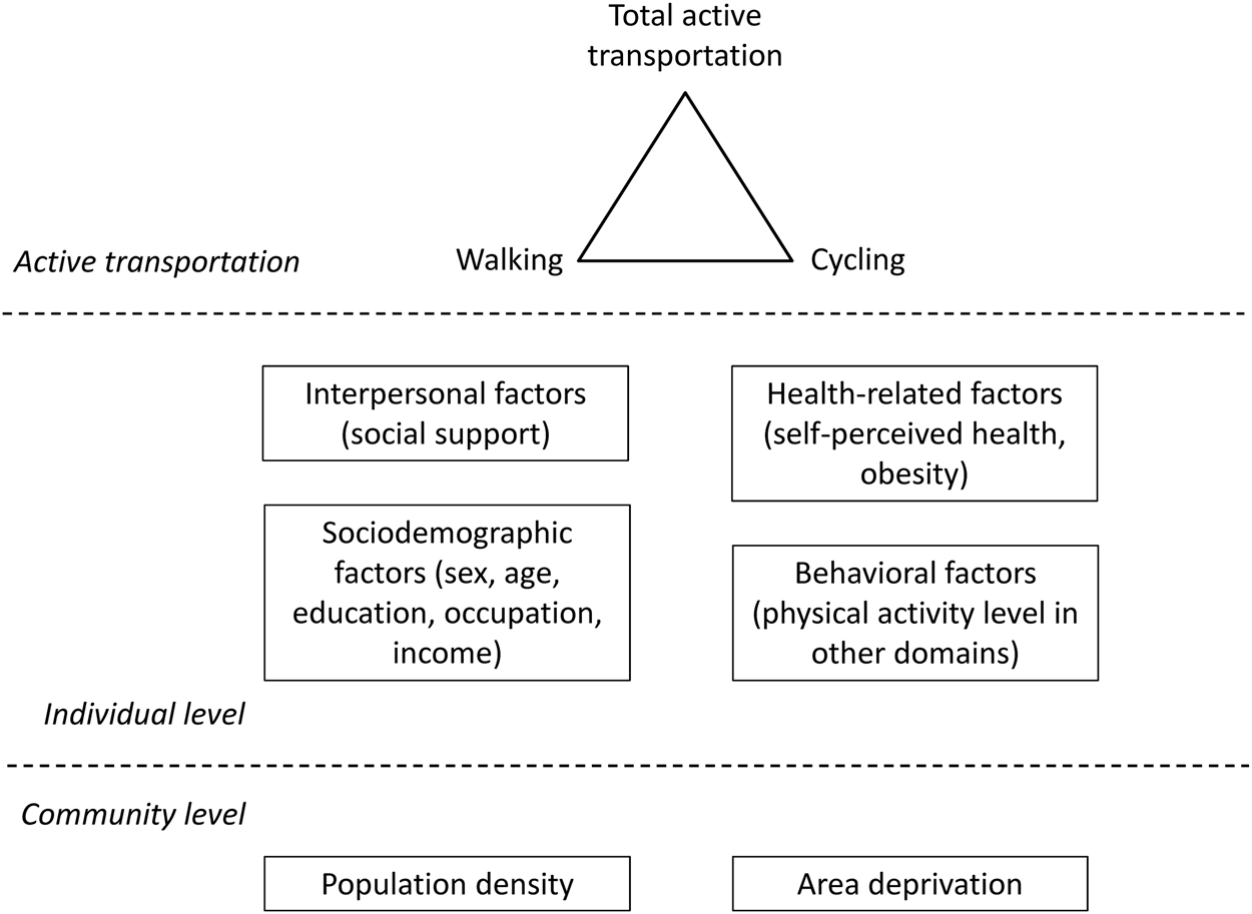
Conceptual framework of individual and community level correlates of active transportation.

*Potential correlates of transportation-related PA* were identified using socioecological models as a conceptual framework and variables were selected based on evidence available in the literature^17–20^. The hypothesized model is shown in Fig. 1. Sex, age, education, occupation, income, social support, work-related and leisure-time PA, self-perceived health, and anthropometric variables were assessed using self-administered questionnaires. *Education* was assessed according to the CASMIN classification of education^21^ based on the highest school and vocational training certificates. A categorical education variable was constructed with ‘low’ (CASMIN 1: primary and low secondary education), ‘medium ‘ (CASMIN 2: intermediate/high secondary education), and high (CASMIN 3: tertiary education) level of education^21^. *Occupational status* and *income* (net household income weighted by household needs) were defined according to the approach published by Lampert *et al*.^22^ and were grouped into tertiles (‘low’, ‘medium’ and ‘high’). *Social support* was assessed according to the 3-item Oslo Social Support Scale, a composite index was calculated and three groups were defined according to the guidelines (‘poor’, ‘medium’ and ‘strong’)^23^. *Work-related* and *leisure-time PA* were assessed with the EHIS-PAQ^9^. In line with the WHO PA recommendations^2^, *leisure-time PA* was grouped into three categories: ‘no activity’,’less than 150 minutes per week’, and ‘at least 150 minutes per week’. Four *work-related PA* categories were used: ‘low - mostly sitting or standing’, ‘medium - mostly walking or moderate physical effort’, ‘high - mostly heavy labor or physically demanding work’ and ‘not working’. *Self-perceived health* was assessed in line with the European Minimum Health Module^24^ and grouped into three categories: ‘bad/very bad’, ‘fair’ and ‘very good/good’. *Obesity* was defined according to the WHO body mass index classification of ≥30 kg/m^2^ ^25^. *Population density* and *area deprivation* were obtained from community-level geographical information and matched to the 301 communities forming the basis of the cluster sample. The methods are described in detail elsewhere^26,27^.

### Study population

The study sample included only GEDA 2014/2015-EHIS participants aged 18 years or older (*n* = 24,016), the reason being that the WHO PA recommendations are defined for the adult population^2^. The final study sample comprised 22,662 persons (10,301 men and 12,361 women) with complete EHIS-PAQ data for walking and cycling; this corresponds to an item response proportion of 94.4%.

#### Statistical analysis

The statistical analyses were performed using the software package STATA SE 14. For all statistical analyses, survey design procedures were used to adjust for the cluster design of the multi-stage sampling procedure. These adjustments lead to wider confidence intervals compared to assuming a simple random sampling. Prevalence and mean values were calculated using a weighting factor that adjusts the sample composition to match the official German population statistics (as of December 31, 2014) according to sex, age, region and education^8^. Binary random-intercept logistic regression models that accounted for the clustering were used to analyze associations between active transportation-related PA recommendation compliance and potential determinants. Population density and area deprivation were used as community level variables; all other variables were used as individual level variables. We used a stepwise procedure. In the first model, we included only the community level variables and adjusted for sex and age. Then, we added the individual level variables (Model 2: +socio-demographic factors, Model 3: +behavioral factors, Model 4: +interpersonal factor, Model 5: +health-related factors) in a stepwise manner. We present the results of the fully-adjusted Model 5, stratified by sex. In the analyses, the following strata were used: age groups: ‘18–29 years’, ‘30–44 years’, ‘45–64 years’, and ‘65+ years’; education, occupation, income, area deprivation, population density, and leisure-time PA: ‘low’, ‘medium’, and ‘high’; work-related PA: ‘low’, ‘medium’, ‘high’ and ‘not working’; obesity: ‘yes/no’; self-perceived health: ‘bad/very bad’, ‘fair’, and ‘very good/good’; social support: ‘poor’, ‘medium’, and ‘strong’. We used the missing-indicator method for data analysis to deal with missing covariate data (missing values are shown in Table 1)^28^. Moreover, we performed a sensitivity analysis using the ‘complete-case method’^29^. Furthermore, the role of sex was analyzed as a potential effect modifier of the associations using a Wald test. The criterion for statistical significance for the interaction analyses was set at p < 0.1.

**Table 1:** Selected variables of the participants aged 18+ years in relation to outcome variables.

	Total sample		Walking ≥600 MET-min/week	Cycling ≥600 MET-min/week	Total active transport ≥600 MET-min/week
	n^a^	%^a^	%^b^ (95% CI)	%^b^ (95% CI)	%^b^ (95% CI)
Total sample	22,662		27.4 (26.5–28.3)	15.1 (14.2–16.2)	40.5 (39.3–41.7)
**Sex**					
men	10,301	45.5	27.0 (25.9–28.1)	17.1 (16.0–18.3)	41.6 (40.2–43.0)
women	12,361	54.5	27.8 (26.6–29.0)	13.2 (12.1–14.4)	39.4 (37.9–40.9)
**Age group (years)**					
18–29	3,835	16.9	25.6 (23.9–27.4)	12.8 (11.3–14.5)	37.8 (35.7–40.1)
30–44	5,223	23.1	22.7 (21.2–24.3)	14.4 (13.0–15.9)	35.7 (33.8–37.7)
45–64	8,610	38.0	25.9 (24.7–27.1)	15.8 (14.6–17.2)	40.5 (39.0–42.1)
65+	4,994	22.0	36.2 (34.5–37.9)	16.7 (15.2–18.3)	47.5 (45.6–49.3)
**Education level**					
low	4,896	21.6	30.1 (28.4–31.7)	14.6 (13.3–16.1)	41.0 (39.2–42.8)
medium	12,279	54.2	26.9 (25.9–28.0)	14.2 (13.1–15.3)	39.3 (37.9–40.8)
high	5,448	24.0	24.1 (22.9–25.4)	19.4 (17.8–21.1)	43.2 (41.3–45.1)
missing	39	0.2			
**Occupation status**					
low	8,544	37.7	27.5 (26.2–28.8)	14.2 (12.9–15.5)	39.1 (37.5–40.8)
medium	6,503	28.7	27.9 (26.5–29.3)	15.0 (13.8–16.3)	40.5 (38.9–42.1)
high	7,586	33.5	26.8 (25.7–28.0)	16.8 (15.4–18.3)	42.5 (40.9–44.1)
missing	29	0.1			
**Income level**					
low	7,194	31.7	29.3 (27.8–30.8)	14.6 (13.3–16.0)	41.4 (39.7–43.1)
medium	7,565	33.4	27.5 (26.1–29.0)	15.2 (14.0–16.4)	40.4 (38.8–42.1)
high	7,903	34.9	25.3 (24.1–26.6)	15.7 (14.5–17.0)	39.5 (37.9–41.2)
**Work-related physical activity**					
low	9,819	43.3	20.1 (19.0–21.1)	14.5 (13.3–15.7)	35.2 (33.6–36.8)
medium	7,629	33.7	30.9 (29.5–32.3)	16.2 (14.9–17.7)	43.9 (42.1–45.6)
high	1,288	5.7	32.7 (29.8–35.7)	12.5 (10.6–14.8)	41.4 (38.4–44.4)
not working	3,926	15.7	34.2 (32.3–36.3)	15.5 (13.9–17.3)	45.1 (42.8–47.3)
missing	367	1.6			
**Leisure-time physical activity**					
low	7,875	34.8	22.2 (21.0–23.6)	7.9 (7.0–8.9)	30.0 (28.3–31.2)
medium	5,477	24.2	22.8 (21.4–24.3)	14.4 (13.0–15.9)	37.6 (35.6–39.6)
high	9,002	39.7	34.8 (33.4–36.1)	22.5 (21.2–24.0)	52.3 (50.7–53.9)
missing	308	1.4			
**Obesity**					
yes	3,759	16.6	24.3 (22.7–25.9)	10.9 (9.6–12.4)	34.8 (32.9–36.7)
no	18,721	82.6	28.1 (27.2–29.0)	16.1 (15.1–17.2)	41.8 (40.5–43.1)
missing	182	0.8			
**Self-perceived health**					
bad/very bad	1,043	4.6	21.2 (18.1–24.6)	7.6 (6.0–9.7)	27.6 (24.3–31.3)
fair	5,445	24.0	27.8 (26.3–29.3)	12.5 (11.2–14.0)	38.2 (36.5–39.9)
very good/good	16,080	71.0	27.7 (26.7–28.7)	16.7 (15.7–17.9)	42.3 (40.9–43.7)
missing	94	0.4			
**Social support**					
poor	3,721	16.4	26.2 (24.3–28.2)	12.4 (11.1–14.0)	37.5 (35.5–39.5)
medium	12,191	53.8	26.9 (25.7–28.1)	14.8 (13.7–15.9)	39.9 (38.5–41.4)
strong	6,453	28.5	29.0 (27.8–30.3)	17.6 (16.1–19.2)	43.6 (41.9–45.4)
missing	297	1.3			
**Population density**					
low	5,474	24.2	26.1 (24.1–28.1)	13.5 (11.5–15.8)	37.6 (35.2–40.0)
medium	11,062	48.8	26.6 (25.5–27.7)	14.0 (12.7–15.3)	38.7 (37.2–40.2)
high	6,126	27.0	30.1 (28.4–31.8)	18.7 (17.0–20.6)	46.3 (44.0–48.6)
**Area deprivation index**					
low	4,815	21.3	26.6 (24.6–28.6)	16.6 (14.5–18.9)	41.3 (38.7–44.1)
medium	12,551	55.4	27.3 (26.2–28.4)	14.6 (13.3–16.0)	40.0 (38.2–41.6)
high	5,296	23.4	28.6 (26.7–30.5)	15.3 (13.5–17.4)	41.3 (39.3–43.3)

^a^Unweighted number of observations/percentages.

^b^Weighted percentages.

### Ethics approval and consent to participate

The study protocol was inspected and approved by the ‘Federal Commissioner for Data Protection and Freedom of Information in Germany’ (No. BfDI: III-401/008#0015). Written informed consent was obtained from all participants. Participants were informed about the goals and contents of the study, about privacy and data protection proceedings, and that their participation in the study was voluntary.

## Results

### Participants, descriptive data, and outcome data

Descriptive characteristics of the study sample stratified by transportation-related PA are provided in Table 1. Prevalence and means of transportation-related outcome data are presented in the Supplementary Information File, Table S1. Amongst the 76.0% of men who indicated engaging in transportation-related walking on at least one day per week, 35.5% spent ≥600 MET-min per week on that activity. Likewise, among the 76.2% of women who reported performing transportation-related walking on at least one day per week, 36.5% spent ≥600 MET-min per week on that activity. Amongst the 32.3% of men who reported engaging in transportation-related cycling on at least one day per week, 52.9% spent ≥600 MET-min per week in that activity. By comparison, among the 27.5% of women who reported performing transportation-related cycling on at least one day per week, 48.0% spent ≥600 MET-min per week on that activity. Amongst the 79.9% of men who indicated that they engaged in transportation-related walking, transportation-related cycling or a combination of transportation-related walking and cycling on at least one day per week, 52.1% spent ≥600 MET-min per week on transportation-related PA. Amongst the 79.5% of women who reported performing transportation-related walking, transportation-related cycling or a combination of transportation-related walking and cycling on at least one day per week, 49.6% spent ≥600 MET-min per week on transportation-related PA.

### Community-level and individual correlates of transportation-related PA

#### Transportation-related walking

In the age- and sex-adjusted community-level Model 1, population density was positively associated with transportation-related walking, with an odds ratio (OR) of 1.29 (1.17–1.44) for high versus low population density. Area deprivation was positively associated with transportation-related walking, with an OR of 1.20 (1.07–1.34) for high versus low area deprivation.

In the fully adjusted Model 5, population density, age, work-related and leisure-time PA, not being obese, and better self-perceived health were all positively associated with transportation-related walking among both men and women (Tables 2 and 3). Area deprivation and social support were positively associated with transportation-related walking among women only. Income level was inversely associated with transportation-related walking in both genders.

**Table 2:** Multivariable-adjusted odds ratios (OR) for correlates of transportation-related walking, cycling and total active transport among men.

Men (n = 10,301)	Walking ≥600 MET-min/week	Cycling ≥600 MET-min/week	Total active transport ≥600 MET-min/week
	OR (95% CI)	OR (95% CI)	OR (95% CI)
***Community level:***			
**Population density**			
low	1.00	1.00	1.00
medium	1.05 (0.94–1.18)	1.02 (0.85–1.23)	1.06 (0.94–1.20)
high	1.20 (1.05–1.38)	1.60 (1.29–1.99)	1.56 (1.35–1.81)
**Area deprivation index**			
low	1.00	1.00	1.00
medium	1.03 (0.91–1.16)	0.96 (0.79–1.17)	1.03 (0.90–1.17)
high	1.15 (0.99–1.33)	1.10 (0.87–1.40)	1.18 (1.01–1.39)
***Individual level:***			
**Age group (years)**			
18–29	1.00	1.00	1.00
30–44	0.89 (0.76–1.04)	1.45 (1.20–1.75)	1.10 (0.96–1.27)
45–64	1.20 (1.04–1.39)	1.51 (1.26–1.79)	1.44 (1.26–1.64)
65+	1.72 (1.46–2.04)	1.68 (1.37–2.06)	1.92 (1.64–2.25)
**Education level**			
low	1.00	1.00	1.00
medium	0.96 (0.86–1.08)	0.92 (0.79–1.07)	0.93 (0.83–1.04)
high	0.94 (0.81–1.09)	1.00 (0.84–1.20)	0.99 (0.86–1.14)
**Occupation status**			
low	1.00	1.00	1.00
medium	1.07 (0.95–1.20)	0.85 (0.74–0.98)	0.98 (0.88–1.09)
high	0.92 (0.81–1.05)	0.92 (0.78–1.07)	0.96 (0.85–1.09)
**Income level**			
low	1.00	1.00	1.00
medium	0.94 (0.84–1.05)	0.94 (0.82–1.08)	0.91 (0.82–1.01)
high	0.85 (0.75–0.96)	0.82 (0.71–0.95)	0.81 (0.72–0.91)
**Work-related physical activity**			
low	1.00	1.00	1.00
medium	1.82 (1.62–2.05)	1.20 (1.05–1.38)	1.53 (1.37–1.71)
high	2.04 (1.72–2.42)	0.95 (0.76–1.18)	1.46 (1.24–1.70)
not working	1.92 (1.65–2.24)	1.27 (1.06–1.53)	1.50 (1.30–1.74)
**Leisure-time physical activity**			
low	1.00	1.00	1.00
medium	1.18 (1.03–1.35)	1.98 (1.68–2.34)	1.62 (1.44–1.83)
high	2.13 (1.91–2.38)	3.52 (3.06–4.05)	2.93 (2.65–3.24)
**Obesity**			
yes	1.00	1.00	1.00
no	1.16 (1.03–1.32)	1.37 (1.17–1.61)	1.26 (1.12–1.41)
**Self-perceived health**			
bad/very bad	1.00	1.00	1.00
fair	1.42 (1.13–1.79)	1.46 (1.06–2.02)	1.56 (1.26–1.93)
very good/good	1.54 (1.23–1.94)	1.91 (1.40–2.62)	1.87 (1.52–2.31)
**Social support**			
poor	1.00	1.00	1.00
medium	1.04 (0.91–1.17)	0.88 (0.76–1.02)	0.94 (0.84–1.06)
strong	1.13 (0.98–1.31)	1.05 (0.89–1.24)	1.07 (0.94–1.22)
ICC	0.0006 (SE 0.0040)	0.0582 (SE 0.0098)	0.0176 (SE 0.0047)

OR: Odds ratio; ICC: Residual Interclass Correlation; SE: Standard Error.

**Table 3:** Multivariable-adjusted odds ratios (OR) for correlates of transportation-related walking, cycling and total active transport among women.

Women (n = 12,361)	Walking ≥600 MET-min/week	Cycling ≥600 MET-min/week	Total active transport ≥600 MET-min/week
	OR (95% CI)	OR (95% CI)	OR (95% CI)
***Community level:***			
**Population density**			
low	1.00	1.00	1.00
medium	1.14 (1.01–1.30)	1.24 (0.96–1.60)	1.20 (1.03–1.39)
high	1.63 (1.41–1.88)	2.38 (1.78–3.20)	2.03 (1.70–2.43)
**Area deprivation index**			
low	1.00	1.00	1.00
medium	1.07 (0.94–1.22)	0.95 (0.73–1.23)	0.99 (0.84–1.16)
high	1.21 (1.03–1.42)	1.28 (0.93–1.77)	1.25 (1.03–1.52)
***Individual level:***			
**Age group (years)**			
18–29	1.00	1.00	1.00
30–44	0.98 (0.85–1.12)	0.99 (0.84–1.18)	0.90 (0.80–1.02)
45–64	1.08 (0.95–1.22)	1.30 (1.11–1.52)	1.14 (1.02–1.28)
65+	1.45 (1.25–1.69)	1.14 (0.93–1.40)	1.26 (1.09–1.46)
**Education level**			
low	1.00	1.00	1.00
medium	1.06 (0.94–1.19)	0.95 (0.81–1.12)	1.06 (0.95–1.19)
high	0.99 (0.84–1.15)	1.24 (1.02–1.52)	1.20 (1.04–1.39)
**Occupation status**			
low	1.00	1.00	1.00
medium	0.99 (0.89–1.11)	0.94 (0.82–1.09)	1.00 (0.91–1.11)
high	0.92 (0.82–1.03)	0.87 (0.75–1.00)	0.97 (0.87–1.07)
**Income level**			
low	1.00	1.00	1.00
medium	0.84 (0.76–0.93)	0.87 (0.76–0.99)	0.82 (0.75–0.91)
high	0.84 (0.75–0.94)	0.79 (0.69–0.92)	0.76 (0.68–0.84)
**Work-related physical activity**			
low	1.00	1.00	1.00
medium	1.71 (1.55–1.89)	1.37 (1.20–1.55)	1.66 (1.52–1.82)
high	3.17 (2.47–4.08)	1.48 (1.08–2.12)	2.35 (1.82–3.02)
not working	2.02 (1.75–2.33)	1.19 (0.98–1.45)	1.80 (1.57–2.07)
**Leisure-time physical activity**			
low	1.00	1.00	1.00
medium	1.07 (0.95–1.20)	1.96 (1.68–2.30)	1.41 (1.27–1.56)
high	1.85 (1.67–2.06)	3.03 (2.62–3.51)	2.35 (2.14–2.59)
**Obesity**			
yes	1.00	1.00	1.00
no	1.28 (1.13–1.44)	1.33 (1.12–1.58)	1.31 (1.17–1.47)
**Self-perceived health**			
bad/very bad	1.00	1.00	1.00
fair	1.55 (1.21–1.97)	1.55 (1.06–2.25)	1.75 (1.40–2.19)
very good/good	1.78 (1.40–2.26)	2.17 (1.50–3.13)	2.24 (1.79–2.80)
**Social support**			
poor	1.00	1.00	1.00
medium	1.06 (0.94–1.20)	1.22 (1.03–1.45)	1.13 (1.00–1.26)
strong	1.17 (1.02–1.34)	1.44 (1.20–1.72)	1.34 (1.18–1.51)
ICC	0.0149 (SE 0.0045)	0.1327 (SE 0.0150)	0.0478 (SE 0.0065)

OR: Odds ratio; ICC: Residual Interclass Correlation; SE: Standard Error.

#### Transportation-related cycling

In the age- and sex-adjusted community-level Model 1, population density was positively associated with transportation-related cycling, with an OR of 1.83 (1.47–2.27) for high versus low population density. Area deprivation was not associated with transportation-related cycling, with an OR of 1.07 (0.84–1.35) for high versus area deprivation.

In the fully adjusted Model 5, in both men and women, population density, leisure-time PA, not being obese, and better self-perceived health were all positively associated with transportation-related cycling, whereas income level was inversely associated with transportation-related cycling (Tables 2 and 3). Education level, social support, age and work-related PA were positively associated with transportation-related cycling among women only.

#### Total active transportation

In the age- and sex-adjusted community-level Model 1, population density was positively associated with total active transportation, with an OR of 1.65 (1.45–1.89) for high versus low population density. Area deprivation was positively associated with total active transportation, with an OR of 1.57 (1.00–1.34) for high versus low area deprivation.

In the fully adjusted Model 5, population density, area deprivation index, age, work-related and leisure-time PA, not being obese, and better self-perceived health were all positively associated with total active transportation in men and women (Tables 2 and 3). Education level and social support were positively associated with total active transportation in women only. Income level was inversely associated with total active transportation in both genders.

### Additional analyses

#### Effect-modification by sex

Compared to men, women showed stronger relations of population density to transportation-related walking (interaction term p-value < 0.001), transportation-related cycling (p-value = 0.029), and total active transportation (p-value = 0.010, Supplementary Information File, Table S2). Men showed stronger relations of age to transportation-related walking (interaction term p-value < 0.058), transportation-related cycling (p value < 0.001) and total active transportation (p-value = 0.002) than women. Women showed positive relations of education level to transportation-related cycling and total active transportation, whereas men exhibited no associations between those variables (interaction term p-values for transportation-related cycling = 0.068; total active transportation = 0.020). Compared to men, women showed stronger relations of work-related PA to transportation-related walking (interaction term p-value = 0.001), transportation-related cycling (p-value = 0.030) and total active transportation (p-value = 0.018). Men showed a stronger association between leisure-time PA and total active transportation than women (interaction term p-value = 0.043). An association between social support and transportation-related cycling was observed only in women but not men (interaction term p-value = 0.035).

#### Sensitivity analysis

Complete-case analyses of the final Models 5 resulted in exactly the same significant associations with comparable effect sizes as presented for the missing indicator method (data not shown).

## Discussion

The present study is the first that examined the prevalence and correlates of complying with the aerobic PA recommendation via transportation-related walking and cycling in the general adult population in Germany. In this large-scale national health interview survey, we observed that 42% of men and 39% of women comply with the minimum level of recommended aerobic PA by a combination of transportation-related walking and cycling. Twenty-seven percent of men and 28% of women achieved PA recommendation compliance with walking, and 17% of men and 13% of women reached the PA recommendation with cycling. Among cyclists, the proportion complying with the recommendation (53% among men and 48% among women) tended to be higher than that among pedestrians (36% among men and 37% among women) because energy expenditure during cycling is significantly higher than during walking^15^.

We identified several potential determinants of transportation-related walking, transportation-related cycling, and total active transportation. High population density, middle or older age, low income, medium-level or high work-related PA, high leisure-time PA, not being obese, and better self-perceived health were consistently positively associated with transportation-related walking, transportation-related cycling, and total active transportation in men and women. Stronger social support and higher level of education were positively associated with active transportation in women only. A high area deprivation index was positively associated with transportation-related walking and total active transportation in women but not with transportation-related cycling.

### Interpretation

Adults who achieve a level of ≥600 MET-min PA per week can expect a reduction of all-cause mortality risk by 30% compared to their insufficiently physically active counterparts^30^. Thus, there is great potential to reduce premature death by promoting active transportation among adults in Germany, since one fifth of the population does not engage in any active transportation and 60% does not achieve the recommended minimum PA level by active transportation activities. Air pollution may reduce the health benefits of active travel, but risk-benefit estimations show that the benefits of transportation-related PA clearly outweigh the risk of air pollution during walking and cycling^31^.

#### Gender differences

In additional analyses, we observed that population density, social support, education level, and work-related PA were more strongly positively associated with transportation-related PA among women compared with men or those relations were limited to women. Safety aspects^18^ may play a role because women feel safer walking or cycling in places with more people on the streets (higher population density) or walking or cycling together as a group (better social support). Our finding that women with a higher education level are more likely to engage in transportation-related cycling than those with a low level of education is in line with study findings from the Netherlands^7^. More research is needed to explore how transportation-related cycling can be increased in the entire population and especially among women with low education background. Also, it remains unclear why the association between work-related PA and transportation-related PA is stronger in women than men.

Higher age and greater leisure-time PA were more strongly positively related to transportation-related PA in men than women. Men showed higher rates of employment (63.9%) than women (54.0%)^32^, and enhanced transportation-related walking and cycling in the newly gained free time when entering retirement age may have a stronger effect in men than women.

#### Age

While older people tend to show less leisure-time PA than younger people^33^, we observed a positive association between age and transportation-related PA, especially among men. Specifically, men aged 65+ years were almost twice as likely to achieve ≥600 MET-min/week through total active transportation than men aged 18 to 29 years. In line with this observation, Fishman *et al*. found positive relations of age to transportation-related walking and cycling among adults in the Netherlands^7^. The resulting health promotion implication is that PA interventions in older people should focus on transportation-related walking and cycling to enhance PA recommendation compliance in this segment of the population because this type of PA is an activity they usually do.

#### Social inequality

Previous studies among adults in Germany observed that higher levels of education, occupation, and income were positively associated with leisure-time PA, whereas they were inversely related to occupational PA^33–36^. A previous study among German adults^37^ showed that education level was positively associated with total active transportation as measured by the Global Physical Activity Questionnaire (GPAQ)^16^, which is in line with our observations among women. Yet, the association between income level and transportation-related PA were consistently inverse among both men and women. Thus, unlike other PA domains, different indicators of socioeconomic position show opposing directions of association in the domain of transportation-related PA. Such an opposing pattern of socioeconomic variables regarding transportation-related PA is noteworthy. Possibly, people with higher income can afford to use a car for transportation purposes, while people with low income may need to walk or cycle to get to and from places because it requires less financial resources. Certain highly-educated, health-conscious women may avoid driving a car to get to and from places and use their bicycles instead to enhance their health and fitness levels and to reduce air pollution and protect the environment^38^.

#### PA in other domains

In a previous study among adults in Germany, we observed that adults with vigorous occupational activity were less likely to engage in sports activity, indicating a strategy of balancing behavior between the PA domains of leisure and work^34^. In the present study, we observed no such pattern between transportation-related PA and other PA domains. Work-related PA and leisure-time PA were consistently positively related to transportation-related walking and cycling, with the exception that men with high work-related PA were as likely to cycle as men with low occupational PA. Thus, people who are more active at work or in leisure time more often choose to use active transportation.

#### Obesity and self-perceived health

In line with findings for other PA domains in previous studies, transportation-related PA was associated with better self-perceived health and a lower prevalence of obesity in our study. Low self-perceived health and obesity have been identified to be barriers to PA^39–41^ but they also may be a consequence of low PA.

#### Social support

The observed positive association between social support and transportation-related PA among women is in line with a recent meta-analysis, consolidating evidence in the literature that social support is positively associated with PA^42^.

#### Area deprivation

Our observation that high area deprivation was positively associated with transportation-related walking and total active transportation among women is in line with the finding that income level was inversely related to transportation-related PA. Women in more deprived neighborhoods may have less financial resources to use a car to get to and from places and they may walk more when using public transportation^43,44^. Also, women living in more deprived neighborhoods may accumulate more walking activities because the distances to get to and from shops are larger than for women living in more affluent neighborhoods with a better infrastructure. Yet, this finding somewhat contradicts study results showing partially positive relations of safe, walkable, and aesthetically pleasing neighborhoods to adult´s physical activity participation^45–47^, assuming that more deprived neighborhoods are generally less safe, less walkable, and aesthetically less pleasing. A study showed that women in the US in states with high compared to low income inequality are less likely to be active in leisure time and to meet PA guidelines^48^. More studies are needed to investigate whether the directions of associations between area deprivation and PA differ by domains of PA.

#### Population density

In population-dense urban places, the average distances to get to shops, workplaces, health care facilities etc. are shorter than in more rural, less population dense places. This could explain why active forms of transportation are more common in urban than rural areas, where a car is needed to get to and from places.

### Measures to increase active transportation behavior

Measures to increase active transportation can be implemented by public, private, and civil stakeholders on several regional levels – from local initiatives in districts or municipalities to international projects. Examples of public initiatives are investments in infrastructure (e.g., bicycle lanes), the foundation of organizations such as the “Pedestrian and Bicycle Information Center”^49^, action plans such as the “German National Cycling Plan”^50^, and international activities such as the project “Cycle Cities”^51^. Initiatives by the private sector include the development and marketing of products that support active transportation (e.g., cycling), business models that address active transportation (e.g., bike sharing) and workplace health promotion activities such as incentives for active commuting. The civil society can promote active transportation through formal organizations like cycling federations or other non-governmental organizations like the initiative “Living Streets”^52^, informal activities like the critical mass movement^53^, or initiatives by private persons or groups (e.g., activities to improve traffic safety in neighborhoods).

The authors of a current systematic review^54^ concluded that research on measures to increase active transportation activities is scant and no clear evidence on the effectiveness of those measures is currently available. Moreover, the risk of bias in existing studies is fairly high. Nevertheless, some intervention studies show that changing environmental components such as reconstructing the infrastructure for walking and cycling have the potential to increase active transportation PA^54,55^. Also, the NICE guideline on walking and cycling^56^ indicates that community-wide promotional activities combined with improved infrastructure has the potential to modestly increase cycling rates.

Currently, there is a large study underway to evaluate determinants and measures to increase active transportation. Specifically, researchers from seven European countries are investigating the effects of active transportation on various outcomes such as health benefits, carbon emission savings, risks of accidents, and health biomarkers. All findings will be integrated in the WHO´s online Health Economic Assessment Tool^57,58^.

### Strength and limitations

An advantage of this national health interview survey is its sophisticated sampling design, ensuring a high degree of generalizability^8^. Other strengths include the high data quality standards applied in the GEDA 2014/2015-EHIS, the high degree of international comparability of the EHIS2 data, and the large sample size of more than 24,000 participants affording ample statistical power and stable results^8,10^. A further asset of the EHIS-PAQ compared to the GPAQ is that the EHIS-PAQ assesses walking and cycling separately^9,16^, which requires less cognitive effort from the participant than having to mentally integrate separate amounts of transportation-related walking and cycling activities when responding to a single question^13,59^.

A potential limitation of the current study is its reliance on self-reported, subjective data, which are prone to a certain degree of reporting bias^13,59^. However, it is difficult if not impossible to objectively assess domain-specific PA information, and the EHIS-PAQ has demonstrated moderate-to-strong reliability and validity for the active transportation items^12^. Estimating MET values from reported walking and cycling rather than directly measuring their energy expenditure is an additional study limitation, which may have resulted in an over- or underestimation of the contribution of active transportation to meeting the PA recommendations. Also, the cross-sectional study design does not allow drawing causal inferences regarding the correlates of active transportation. Selection bias may have occurred because the response rate was only 27.6%. However, a comparison between the study sample distribution of GEDA 2014/15-EHIS and the German population structure (German Statistical Office 2014) according to selected characteristics indicates that the representativeness of our sample is high and that the adaptive weighting factor corrections are small^8^. Another limitation of the study is that other potential influencing factors for transportation-related walking and cycling such as ‘distance to the workplace’^60^ were not considered because no such data were available. In this study, we assessed whether individuals meet recommended PA levels only with transportation-related PA regardless of PA in other domains (e.g., leisure-time or work-related PA). Thus, it would be interesting to quantify the number of persons who are meeting PA recommendations from work-related and leisure-time PA domains and then identify the additional number of persons who would meet recommendations if transportation-related PA were included.

## Conclusions

We conclude that promoting transportation-related walking and cycling among inactive people has great potential to increase PA recommendation compliance in the general population. We identified several potential determinants of active transportation that should be considered when developing public health policies and carrying out activity interventions. It is noteworthy that the elderly and individuals with lower levels of income appear to be more likely to achieve PA recommendations compliance through transportation-related activities; those population subgroups typically show lower participation in other PA domains, including leisure-time PA. Additional investments in pedestrian and cycling infrastructure are needed to increase the share of the population willing to engage in transportation-related activity in the future. Increasing the levels of transportation-related activity is important from a public health perspective but also from an ecological point of view because it can help combat climate change, reach the targets of the Paris climate agreement, and reduce air pollution in urban places.

## Supplementary information

Supplementary Information File

## Data Availability

The datasets supporting the conclusions of this article are available via Public Use File, Data Use Agreement or can be accessed on site upon reasonable request at the Research Data Centre of the Robert Koch Institute (https://www.rki.de/DE/Content/Forsch/FDZ/FDZ_node.html). The Research Data Centre is accredited by the German Data Forum according to uniform and transparent standards.
