# Supplementary material for: Area-level and individual correlates of active transportation among adults in Germany: A population-based multilevel study
Source: Sci Rep. 2019 Nov 8;9:16361. doi: 10.1038/s41598-019-52888-x (PMC6841943; doi:10.1038/s41598-019-52888-x)
Supplement: Supplementary file 1 — Supplementary Information File [file 41598_2019_52888_MOESM1_ESM.pdf]

Supplementary Information File

Area-level and individual correlates of active transportation among adults in Germany: A population-based multilevel study

Finger, J.D., Varnaccia, G., Gabrys, L., Hoebel, J., Kroll, L.E., Krug, S., Manz, K., Baumeister, S.E., Mensink, G.B.M., Lange, C., Leitzmann, M.F.

Table S1: Prevalence/ Means of transportation-related physical activity (TRPA) outcome data			
	**Walking**	**Cycling**	**Walking and cycling combined**
	%/Mean (95% CI)	%/Mean (95% CI)	%/Mean (95% CI)
**TRPA of less than one day per week in %**			
Men	24.0 (22.9-25.2)	67.7 (66.0-69.3)	20.1 (19.0-21.3)
Women	23.8 (22.5-25.1)	72.5 (70.6-74.3)	20.5 (19.3-21.8)
**TRPA mean minutes per week**			
Men	180.1 (173.6-186.7)	50.8 (47.3-54.2)	-
Women	178.6 (172.0-185.3)	36.6 (33.4-39.7)	-
**TRPA mean MET-minutes per week [a]**			
Men	594.4 (572.8-616.1)	304.7 (283.9-325.5)	899.1 (865.4-932.9)
Women	589.4 (567.5-611.4)	219.3 (200.5-238.1)	808.8 (777.3-840.2)
[a] The metabolic equivalent (MET) values used for computing MET-minutes were 3.3 for walking and 6.0 for cycling			

Table S2: Odds ratios (OR) of interaction terms of sex*exposure variables to transport-related physical activity outcome variables			
	**Walking ≥ 600 MET-min/week**	**Cycling ≥ 600 MET-min/week**	**Total active transport ≥ 600 MET-min/week**
	OR (95% CI)	OR (95% CI)	OR (95% CI)
**Population density**			
Sex*medium versus low	1.09 (0.94-1.26)	1.15 (0.95-1.38)	1.12 (0.97-1.28)
Sex*high versus low	1.34 (1.14-1.58)	1.31 (1.07-1.60)	1.26 (1.09-1.47)
Overall p-values (Wald test)	0.0008	0.0286	0.0097
**Area deprivation index**			
Sex*medium versus low	0.95 (0.82-1.10)	0.94 (0.79-1.13)	0.91 (0.79-1.05)
Sex*high versus low	0.88 (0.74-1.05)	1.01 (0.82-1.25)	0.96 (0.82-1.13)
Overall p-values (Wald test)	0.3556	0.6767	0.3819
**Age group (years)**			
Sex*30-44 versus18-29	1.20 (0.98-1.46)	0.78 (0.61-0.99)	0.95 (0.79-1.13)
Sex*45-64 versus 18-29	1.04 (0.87-1.24)	1.03 (0.83-1.28)	0.96 (0.82-1.13)
Sex*65+ versus 18-29	0.94 (0.78-1.13)	0.69 (0.54-0.87)	0.75 (0.63-0.90)
Overall p-values (Wald test)	0.0582	0.0001	0.0024
**Education level**			
Sex*medium versus low	1.14 (0.99-1.32)	1.23 (1.02-1.48)	1.24 (1.08-1.43)
Sex*high versus low	1.08 (0.91-1.29)	1.30 (1.06-1.61)	1.20 (1.02-1.41)
Overall p-values (Wald test)	0.1632	0.0681	0.0202
**Occupational status**			
Sex*medium versus low	0.88 (0.76-1.02)	1.07 (0.90-1.29)	0.98 (0.86-1.12)
Sex*high versus low	0.95 (0.83-1.10)	0.98 (0.83-1.17)	0.98 (0.86-1.12)
Overall p-values (Wald test)	0.3974	0.6061	0.7984
**Income level**			
Sex*medium versus low	0.91 (0.79-1.06)	0.91 (0.76-1.08)	0.91 (0.79-1.04)
Sex*high versus low	1.08 (0.93-1.24)	0.97 (0.81-1.15)	0.97 (0.85-1.10)
Overall p-values (Wald test)	0.0810	0.5497	0.3449
**Work-related physical activity**			
Sex*medium versus low	0.85 (0.74-0.98)	0.99 (0.84-1.16)	0.97 (0.85-1.10)
Sex*high versus low	1.49 (1.12-1.98)	1.34 (0.91-1.99)	1.52 (1.15-2.01)
Sex*not working versus low	0.89 (0.75-1.05)	0.76 (0.61-0.94)	0.96 (0.82-1.12)
Overall p-values (Wald test)	0.0011	0.0303	0.0177
**Leisure-time physical activity**			
Sex*medium versus low	0.96 (0.81-1.14)	1.05 (0.84-1.31)	0.89 (0.77-1.04)
Sex*high versus low	0.91 (0.80-1.05)	0.88 (0.73-1.06)	0.83 (0.73-0.95)
Overall p-values (Wald test)	0.3093	0.1105	0.0428
**Obesity**			
Sex*no versus yes	1.11 (0.94-1.31)	0.96 (0.77-1.19)	1.05 (0.90-1.21)
p-value	0.206	0.692	0.560
**Self-perceived health**			
Sex*fair versus bad/very bad	1.09 (0.80-1.50)	1.12 (0.70-1.80)	1.06 (0.79-1.43)
Sex*very good/good versus bad/very bad	1.23 (0.91-1.67)	1.28 (0.82-2.02)	1.22 (0.92-1.62)
Overall p-values (Wald test)	0.2194	0.3881	0.1179
**Social support**			
Sex*medium versus poor	0.99 (0.84-1.17)	1.32 (1.07-1.64)	1.15 (0.98-1.34)
Sex*strong versus poor	0.98 (0.81-1.17)	1.32 (1.05-1.66)	1.18 (0.99-1.39)
Overall p-values (Wald test)	0.9957	0.0345	0.2030
